# Evaluation of drug interaction potential of *Labisia pumila* (Kacip Fatimah) and its constituents

**DOI:** 10.3389/fphar.2014.00178

**Published:** 2014-08-08

**Authors:** Vamshi K. Manda, Olivia R. Dale, Charles Awortwe, Zulfiqar Ali, Ikhlas A. Khan, Larry A. Walker, Shabana I. Khan

**Affiliations:** ^1^National Center for Natural Products Research, School of Pharmacy, The University of MississippiOxford, MS, USA; ^2^Division of Clinical Pharmacology, University of StellenboschCape Town, South Africa; ^3^Division of Pharmacognosy, Department of Biomolecular Sciences, School of Pharmacy, The University of MississippiOxford, MS, USA; ^4^Division of Pharmacology, Department of Biomolecular Sciences, School of Pharmacy, The University of MississippiOxford, MS, USA

**Keywords:** *Labisia pumila*, Myrsinaceae, herb-drug interactions, PXR, CYP450 enzymes, P-gp

## Abstract

*Labisia pumila* (Kacip Fatimah) is a popular herb in Malaysia that has been traditionally used in a number of women’s health applications such as to improve libido, relieve postmenopausal symptoms, and to facilitate or hasten delivery in childbirth. In addition, the constituents of this plant have been reported to possess anticancer, antioxidant, and anti-inflammatory properties. Clinical studies have indicated that cytochrome P450s (CYPs), P-glycoprotein (P-gp), and Pregnane X receptor (PXR) are the three main modulators of drug-drug interactions which alter the absorption, distribution, and metabolism of drugs. Given the widespread use of Kacip Fatimah in dietary supplements, the current study focuses on determining the potential of its constituents to affect the activities of CYPs, P-gp, or PXR using *in vitro* assays which may provide useful information toward the risk of herb-drug interaction with concomitantly used drugs. Six compounds isolated from the roots of *L. pumila* (2 saponins and 4 alkyl phenols) were tested, in addition to the methanolic extract. The extract of *L. pumila* showed a significant time dependent inhibition (TDI) of CYP3A4, reversible inhibition of CYP2C9 and 2C19 and a weak inhibition of 1A2 and 2D6 as well as an inhibition of P-gp and rifampicin-induced PXR activation. The alkyl phenols inhibited CYP3A4 (TDI), CYP2C9, and 2C19 (reversible) while saponins inhibited P-gp and PXR. In conclusion, *L. pumila* and its constituents showed significant modulation of all three regulatory proteins (CYPs, P-gp, and PXR) suggesting a potential to alter the pharmacokinetic and pharmacodynamic properties of conventional drugs if used concomitantly.

## INTRODUCTION

*Labisia pumila* (Blume) Fern.-Vill., locally termed as Kacip Fatimah (KF), is a popular herb in South East Asian countries. It belongs to the Myrsinaceae family. Recently it has been identified as one of the top five herbs used in Malaysia for treating variety of ailments (Karimi et al., 2013). Traditionally, KF is mainly used in a wide spectrum of women’s health related issues; the effects are presumed as attributable to the presence of estrogen-like compounds. KF is often taken during and after pregnancy for its beneficial effects on uterine function and delivery. The primary route of administration of KF is oral, whereby the leaves, roots, or whole plant are boiled in water and consumed. Additionally, it is sold commercially in the form of herbal tea, powder, capsules, and tablets in many countries (Abdul Kadir et al., 2012). Clinical studies have also suggested the usefulness of KF extract in treating postmenopausal symptoms (Abdul Kadir et al., 2012) with no acute toxicity (Singh et al., 2009). Furthermore, the extract and constituents of KF have been shown to possess anticancer, antioxidant, anti-osteoporosis, and anti-inflammatory properties (Nadia et al., 2012; Fathilah et al., 2013).

Due to the increasing popularity and wide spread use of herbal supplements throughout the world, there is a potential risk of herb-drug interactions when these supplements are taken in combination with conventional drugs, as there is often limited standardization of dose of herbal supplements taken. This is evident by the increasing reports of clinical cases of toxicity caused by herb-drug interactions (Chen et al., 2011, 2012). Early identification of drug interaction potential of herbal supplements and their constituents will aid in lowering the risk of herb-drug interactions. It is widely documented that CYPs, P-gp, and PXR are the three main modulators of drug-drug interactions as these are involved in affecting the pharmacokinetic and pharmacodynamic properties of xenobiotics (Alissa, 2014). Despite the use of KF as a herbal medicine, limited studies exist in literature for its drug interaction potential. A recent study has indicated that different extracts of *L. pumila* show potent inhibition of CYPs, specifically CYP2C isoforms (Pan et al., 2012). However, there are no studies identifying the chemical constituents of KF responsible for CYP inhibition. As part of our phytochemical studies on medicinal plants, several constituents have been isolated from the roots of *L. pumila*; these belong to various chemical classes, including saponins, alkyl phenols, cerebroside, glycerogalactolipids, and lipids (Ali and Khan, 2011).

In the extension of these studies, the current investigation focuses on determining the potential of KF methanolic extract and its constituents to affect the activities of major drug metabolizing enzymes (CYP 3A4, 2D6, 1A2, 2C9, and 2C19), P-gp, and PXR using *in vitro* assays which may provide useful information toward the risk of herb-drug interactions with concomitantly used drugs. The inhibition of CYP 3A4, 2D6, 1A2, 2C9, and 2C19 was determined by employing C-DNA baculovirus expressed recombinant enzymes and specific fluorescent substrates. The inhibition of P-gp was determined in hMDR1-MDCK-II (Madin-Darby canine kidney) and MDCK-II cells by using two widely used substrates calcein-AM and digoxin. Modulation of PXR activity was monitored through a reporter gene assay in HepG2 cells transfected with PXR plasmid DNA and a luciferase reporter plasmid PCR5. Additionally, we used FDA guided assumptions (Zhang et al., 2009) to predict the likelihood of the KF extract and its constituents to cause herb drug interactions (HDI) *in vivo*.

## MATERIALS AND METHODS

Madin-Darby canine kidney-II (parental) and hMDR1-MDCK-II (transfected) cell lines were a gift from Dr. Gottesman (NIH, Bethesda, USA). Dulbecco’s Modified Eagle Medium (DMEM), Minimal Essential Medium (MEM), Hanks balanced salt solution (HBSS), HEPES, Trypsin EDTA, Penicillin-streptomycin, and Sodium Pyruvate were from GIBCO BRL (Invitrogen Corp., Grand Island, NY, USA). Fetal bovine serum (FBS) was from Hyclone Lab Inc. (Logan, UT, USA). CYP1A2/CEC, CYP2C9/MFC, CYP3A4/BQ, CYP2C19/CEC, and CYP2D6/AMMC high throughput inhibitor screening kits were from BD Gentest (Woburn, MA, USA). Transwell plates (12 mm diameter, 0.4 μM pore size) were from Costar Corp. (Cambridge, MA, USA). All other chemicals were from Sigma Chem. Co., (St. Louis, MO, USA). Radio labeled digoxin [^3^H-digoxin, 0.25 mCi/0.25 ml] was from Perkin Elmer Life Sciences (Waltham, MA, USA). Troleandomycin was from Santa Cruz Biotechnology, Inc. (Dallas, TX, USA). Preparation of *L. pumila* methanolic extract and isolation of its constituents used in the current study were described in our previous study (Ali and Khan, 2011).

### CULTURE OF hMDR1-MDCK-II, MDCK-II AND HepG2 CELLS

Parental and transfected MDCK-II cells were grown in DMEM supplemented with 10% FBS, 1% non-essential amino acids, 1% L-glutamine, 100 U/ml penicillin-G, and 100 μg/ml streptomycin at 37°C, 95% relative humidity, and 5% CO_2_. Cells were seeded at a density of 65,000 cells/well (0.5 mL) on the apical side of a 12-well Transwell plate and 1.5 ml of medium was added to the basolateral side. HepG2 cells were grown in DMEM/F12 medium supplemented with 10% FBS, 2.4 g/L sodium bicarbonate, 100 U/ml penicillin-G, and 100 μg/ml streptomycin at 37°C, 95% relative humidity, and 5% CO_2_.

### ASSAYS FOR REVERSIBLE INHIBITION (CO-INCUBATION ASSAY) AND TIME DEPENDENT INHIBITION (PRE-INCUBATION ASSAY) OF CYPs

The assay for reversible inhibition was conducted in a total volume of 200 μL in 96-well microplates. The assay conditions, enzyme and substrate concentrations were similar as reported earlier (Crespi et al., 1997; Manda et al., 2013). Test samples or positive controls were serially diluted in a solution (100 μL) of cofactors mix, control protein (0.05 mg of protein/mL), and G-6-PDH to achieve six concentrations (100-0.4 μM or μg/mL). Initial readings were taken to record any inherent fluorescence and the plates were incubated at 37°C for 10 min. Reaction was initiated by the addition of enzyme substrate mixture (100 μL) followed by incubation for 15, 30, or 45 min. The reaction was terminated by the addition of 75 μL of ice cold acetonitrile/0.5 M Tris base (80:20). Fluorescence was measured on Spectramax M5 plate reader (Molecular Devices, Sunnyvale, CA, USA) at specified excitation and emission wavelengths for each substrate. IC_50_ values (co-incubation assay) were obtained from concentration-response curves generated by plotting concentration versus % inhibition.

Time dependent inhibition (TDI) of CYPs was measured as described earlier by Sekiguchi et al. (2009). The reaction mixture (180–190 μL), consisting of test sample, recombinant enzyme, control protein (0.05 mg of protein/mL), cofactor mix, G-6-PDH, and 50 mM potassium phosphate buffer (pH 7.4) was pre-incubated for 30 min followed by addition of respective fluorescent substrates (10–20 μL) and further incubation for 15 (CYP1A2), 30 (CYP3A4), or 45 (CYP 2C9 and 2C19) min. The reaction was terminated by addition of 75 μL of ice cold acetonitrile/0.5 M Tris base (80:20) and fluorescence was measured as above. IC_50_ values (pre-incubation assay) were obtained as above. The shift in the concentration-response curve was calculated as the ratio of IC_50_ (co-incubation)/IC_50_ (pre-incubation).

### ASSAY FOR PXR MODULATION

The pSG5-hPXR expression vector was provided generously by Dr. Steven Kliewer (University of Texas Southwestern Medical Center, Dallas, TX, USA; Lehmann et al., 1998) and the reporter plasmid CYP3A4-PXR response element (PXRE)-LUC (containing the proximal 0/–362 and distal 7208/7797 PXRE regions fused upstream of luciferase; Goodwin et al., 1999) was a kind gift from Dr. Christopher Liddle (University of Sydney, Westmead, NSW, Australia). The modulation of PXR activity by test samples was determined in HepG2 cells transiently transfected with pSG5-PXR (25 μg) and PCR5 plasmid DNA (25 μg) by electroporation at 180 V, 1 pulse for 70 msec. The cells were plated in 96-well plates at a density of 50,000 cells per well. After 24 h, test samples and drug controls were added at various concentrations. After additional 24 hour incubation, the media was aspirated and 40 μL of luciferase reagent (Promega Corporation, Madison, WI, USA) was added to each well and luminescence was measured on Spectramax M5 plate reader (Molecular Devices, Sunnyvale, CA, USA). The fold induction in luciferase activity in the treated cells was calculated in comparison to vehicle treated cells. The cytotoxicity of test samples toward HepG2 cells was also determined by measuring the cell viability using the CellTiter 96 AQ_ueous_ One Solution Cell Proliferation Assay (MTS) as described earlier (Manda et al., 2013).

### ASSAY FOR P-gp INHIBITION BY CALCEIN-AM UPTAKE IN PARENTAL AND TRANSFECTED MDCK-II CELLS

The assay was performed as described previously (Rautio et al., 2006). Cells were seeded in 96-well plates at 70,000 cells/well in 200 μL of culture medium. The medium was changed at 24 h after seeding and the assay was performed 48 h later. Test samples at various concentrations and positive control (verapamil 100-0.4 μM) were added to the cells in 50 μL of transport buffer and incubated at 37°C for 10 min. Calcein-AM (1 μM), a fluorescent P-gp substrate) was added and the plates were immediately placed on Spectramax and fluorescence was read up to 1 h at 15-min intervals at excitation and emission wavelengths of 485 and 530 nm, respectively. The % increase in calcein-AM uptake was calculated as described earlier (Liu et al., 2008; Manda et al., 2014).

The EC_50_ value, defined as the concentration that caused an increase of 50% in calcein-AM uptake, was obtained from dose curves generated by plotting % increase in calcein-AM uptake versus log concentration using GraphPad Prism.

### ASSAY FOR P-gp INHIBITION BY ^3^H-DIGOXIN UPTAKE IN hMDR1-MDCK-II CELLS

The assay conditions were similar as described earlier (Rautio et al., 2006) with some modifications. The cells were seeded at a density of 120,000 cells/well in 12-well Transwell plates and cultured for 3 days. TEER values were in the range of 500–800 Ω cm^2^. Cells were washed with warm HBSS buffer supplemented with 10 mM HEPES (pH 7.4) and pre-incubated with 0.5 mL of buffer containing test samples (six concentrations) on the apical side and 1.5 mL of buffer on the basolateral side for 30 min (37^o^C, 5% CO_2_, and 95% relative humidity). After incubation, buffer was removed from the basolateral side and replaced with 1.5 mL of buffer containing ^3^H-digoxin (40 nM), test compounds or standard drugs (25 μM), and incubated further for 2 h. Aliquots of 25 μL were taken out from the apical side, mixed with 100 μL of scintillant (Microscint TM-40, PerkinElmer) and radioactive counts were measured on a TopCount microplate scintillation counter (PerkinElmer, Waltham, MA, USA) in CPM mode. The monolayer integrity was monitored by measuring the permeability of Ly (a fluorescent marker of passive paracellular diffusion) as described earlier (Manda et al., 2013).

The inhibition of the basolateral to apical (B-A) transport of digoxin by test samples was calculated compared to the vehicle control. The IC_50_ value, defined as the concentration that caused an inhibition of 50% in digoxin transport, was obtained from dose curves generated by plotting % inhibition versus log concentration using GraphPad Prism.

### PREDICTION OF *IN VIVO* HDI FROM *IN VITRO* RESULTS

All assumptions to predict the HDI potential of KF methanolic extract and its constituents were according to previously published report (Awortwe et al., 2014). The % yield was calculated from the amounts extracted from the KF roots (Ali and Khan, 2011). The human GIT volume is 250 mL and plasma volume is about 3 L. The commonly used maximum dose of KF extract capsules is 560 mg per day (Abdul Kadir et al., 2012) and accordingly we estimated the concentration per dose, GIT, and plasma concentrations of extract and its constituents. We then compared the *in vitro* IC_50_ values obtained from recombinant CYPs with estimated GIT and plasma concentrations. If the IC_50_ values were lower than the GIT or plasma concentration, then the test compound or extract is likely to cause HDI *in vivo.* The prediction was not done for two saponins as they did not show any inhibition toward CYPs tested.

### STATISTICAL METHODS

All values are represented as mean ± SD (*n* = 3). The data were analyzed by one way ANOVA, followed by Dunnett’s multiple comparison tests using GraphPad Prism Version 5, (San Diego, CA, USA). *P* < 0.05 was considered to be statistically significant.

## RESULTS

### REVERSIBLE INHIBITION AND TDI OF CYPs

**FIGURE 1:**
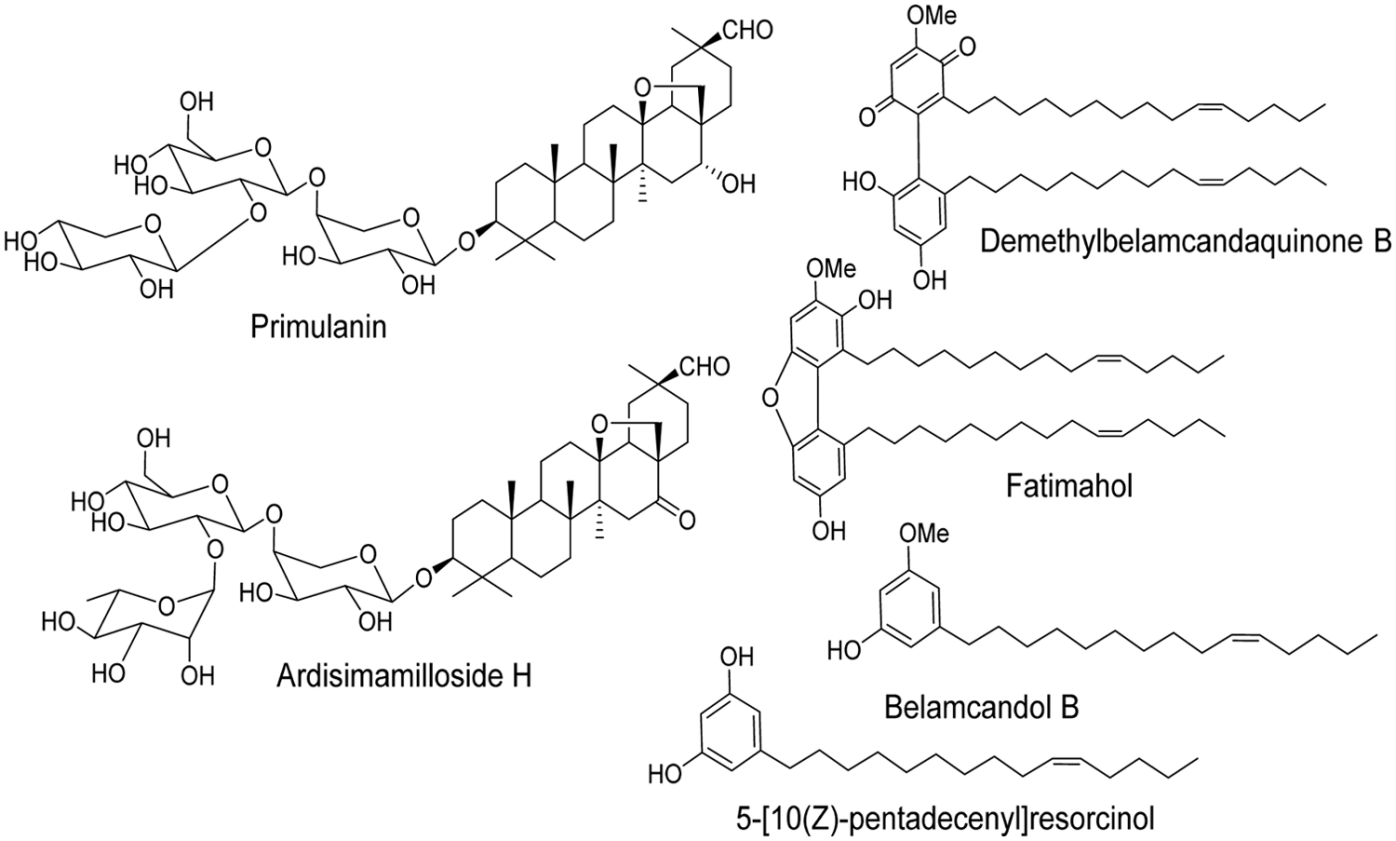
**Chemical structures of two saponins and four alkyl phenols isolated from the roots of *Labisia pumila***.

The two major classes of compounds isolated from the roots of KF are alkyl phenols and triterpene glycosides (saponins) as reported earlier (Ali and Khan, 2011). We have determined the effect of methanolic extract of KF and its six constituents (4 alkyl phenols and 2 saponins, **Figure 1** on major CYPs using specific fluorescent substrates and recombinant enzymes.

**FIGURE 2:**
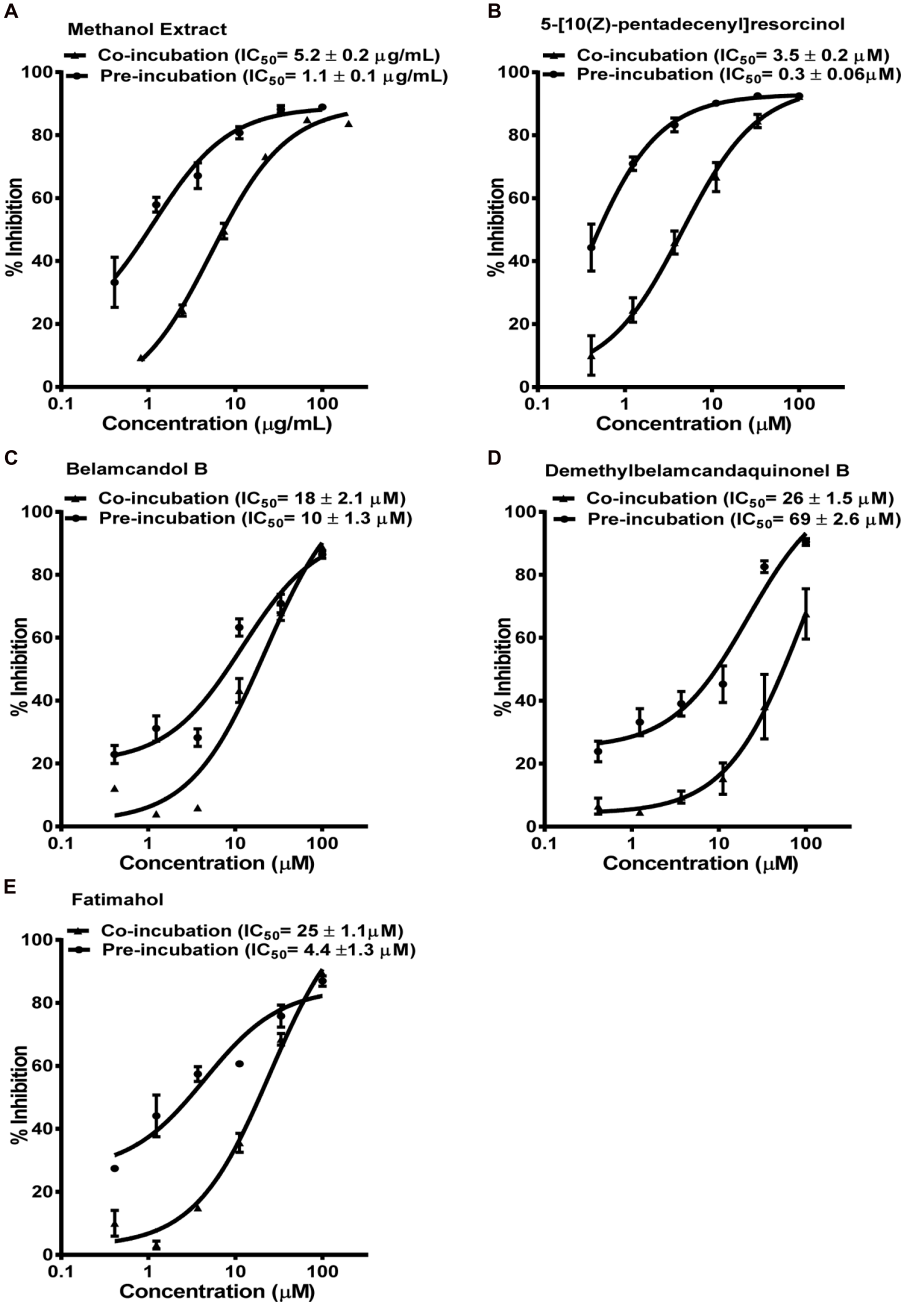
**Dose response profiles of reversible and time dependent inhibition (TDI) of CYP3A4 enzyme by *Labisia pumila* root extract **(A)** and its alkyl phenolic constituents **(B–E)**.** The data are represented as mean ± SD of 3 independent experiments (*n* = 2 in each experiment).

**FIGURE 3:**
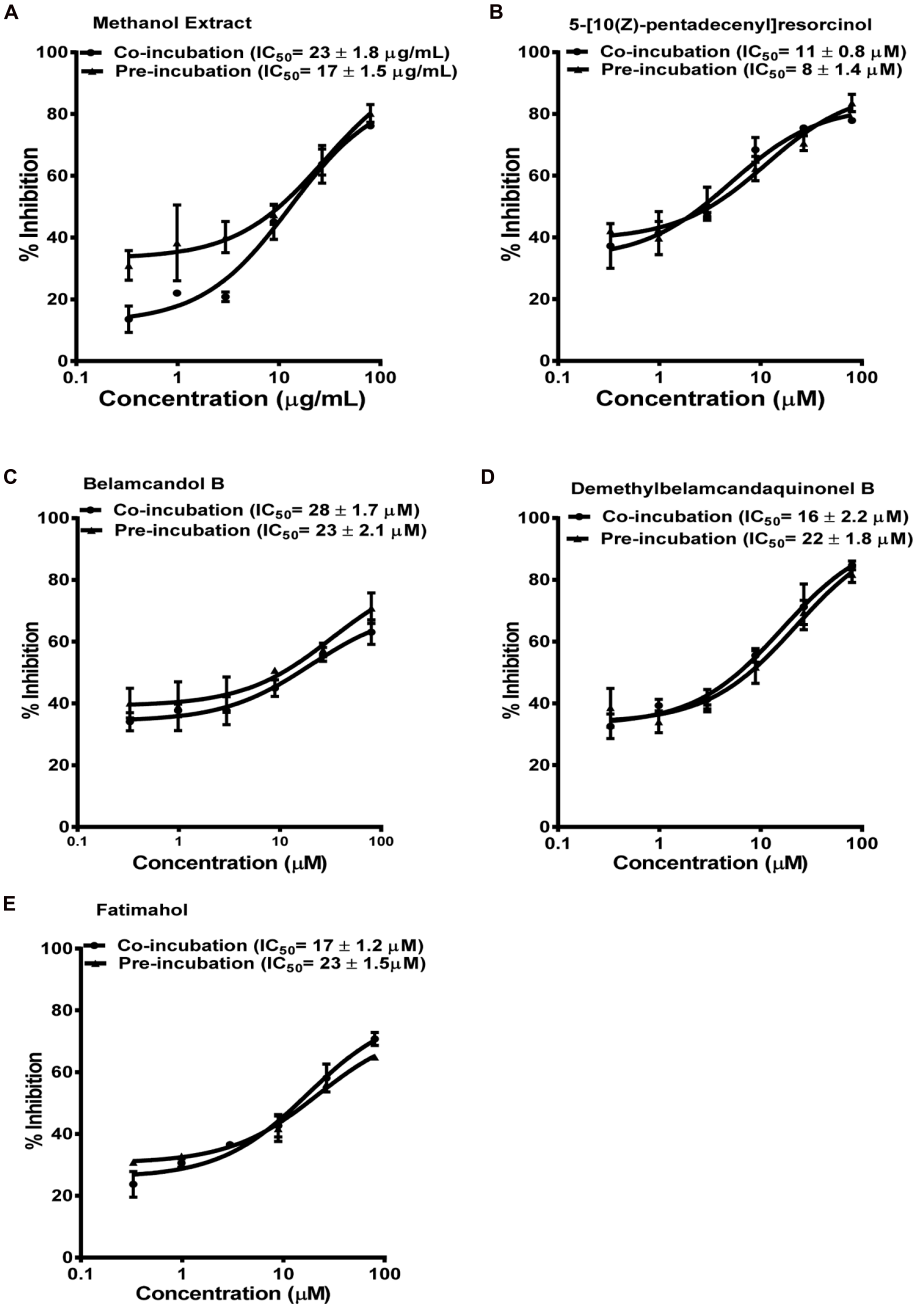
**Dose response profiles of reversible and TDI of CYP2C9 enzyme by *Labisia pumila* root extract **(A)** and its alkyl phenolic constituents **(B–E)**.** The data are represented as mean ± SD of 3 independent experiments (*n* = 2 in each experiment).

**FIGURE 4:**
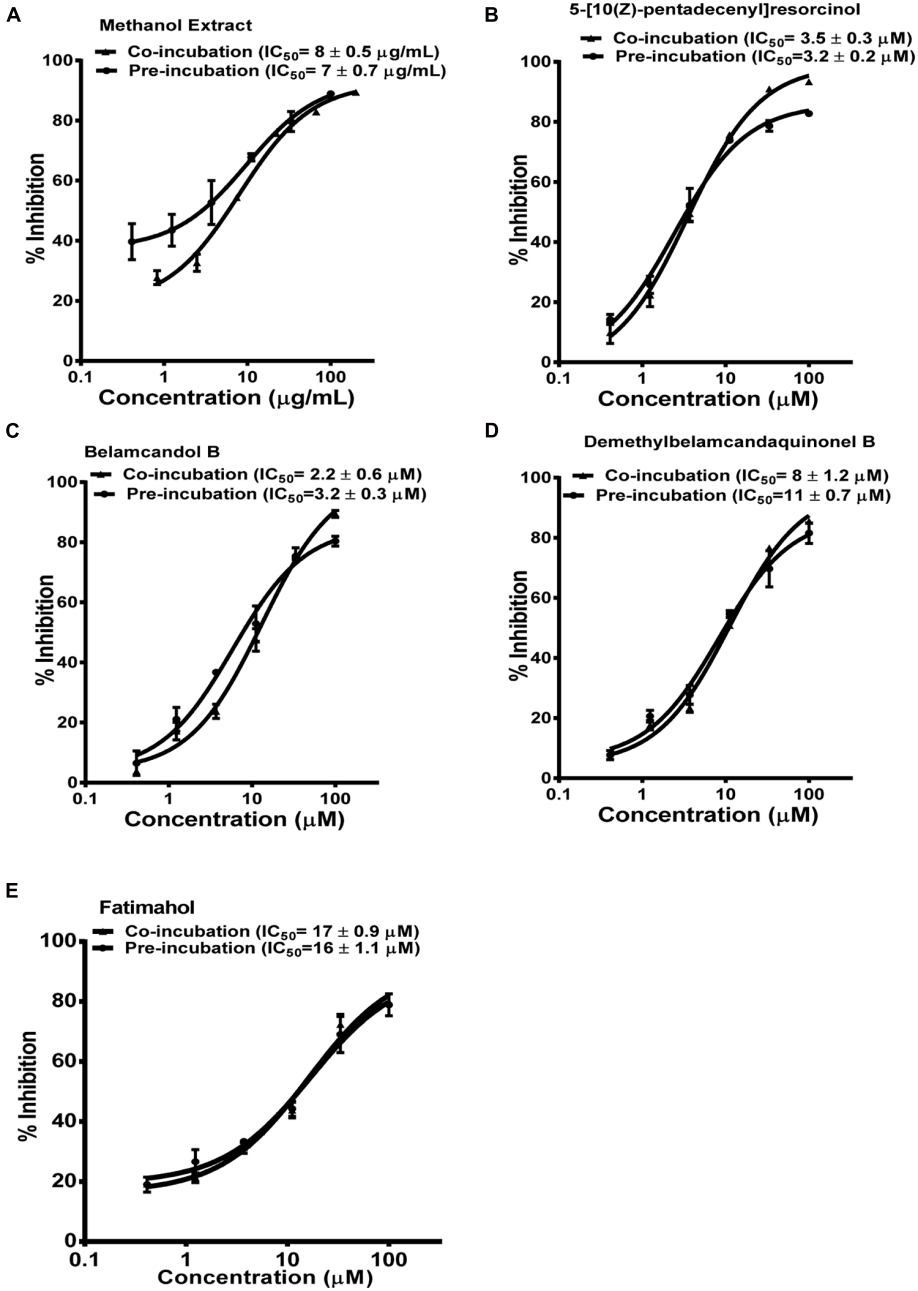
**Dose response profiles of reversible and TDI of CYP2C19 enzyme by *Labisia pumila* root extract **(A)** and its alkyl phenolic constituents **(B–E)**.** The data are represented as mean ± SD of 3 independent experiments (*n* = 2 in each experiment).

The methanolic extract and alkyl phenolic compounds showed dose dependent inhibition of CYP3A4, 2C9, and 2C19 (**Figures 2–4**) while saponins did not affect the activity of these enzymes. Out of the alky phenols, 5-[10(Z)-pentadecenyl]-resorcinol was effective in inhibiting CYP3A4 and 2C9 with IC_50_ values of 4.1 ± 0.2 and 11 ± 0.8 μM, respectively, while belamcandol B inhibited CYP2C19 with an IC_50_ value of 2.2 ± 0.6 μM.

Based on these results, we further tested the TDI potential of extract and selected constituents toward CYP3A4, 2C9, and 2C19. The test samples were pre-incubated for 30 min with the co-factors, control protein, and specific enzymes before substrates were added. IC_50_ shift was determined as described in the Materials and Methods. Compounds which showed IC_50_ shift ratio of greater than 1.5 were considered to have potential to exhibit TDI. Based on these criteria, no time-dependent inhibition was observed with recombinant CYP2C9 and 2C19 enzymes by the test compounds or the methanol extract. The dose curves were identical from co-incubation and pre-incubation experiments (**Figures 3 and 4**. The IC_50_ shift fold ratios of the control drugs (tranylcypromine, **Table 1**) were similar to the published literature values (Naritomi et al., 2004). In contrast, the methanol extract as well as the four alkyl phenols showed a very potent TDI of CYP3A4 with the dose curves shifted significantly to the left, as shown in **Figure 2**. The IC_50_ shift fold ratio for 5-[10(Z)-pentadecenyl]-resorcinol, fatimahol, and methanol extract was 13.6, 5.6, and 4.7, respectively, (**Table 1** suggesting a very strong potential for TDI of CYP3A4 by these agents. The positive control for TDI, troleandomycin, showed an IC_50_ shift fold ratio of 3.4 **Table 1** which is in accordance to the previous report (Sekiguchi et al., 2009). Further, the dose response curves clearly indicate a significant increase in % inhibition when the extract or the compounds were preincubated with CYP3A4 enzyme (**Figure 2**).

**Table 1:** IC_**50**_ of CYP3A4 inhibition by *Labisia pumila* methanol extract and its isolated constituents.

	CYP3A4		
Extract/compound	IC_50_ (μM) Co-incubation	IC_50_ (μM) Pre-incubation	IC_50_ Shift (fold)
Methanol extract (μg/mL)	5.2 ± 0.2	1.1 ± 0.1	4.7
5-[10(Z)-pentadecenyl]resorcinol	4.1 ± 0.2	0.3 ± 0.06	13.6
Belamcandol B	18 ± 2.1	10 ± 1.3	1.8
Demethylbelamcandaquinone B	69 ± 2.6	26 ± 1.5	2.6
Fatimahol	25 ± 1.1	4.4 ± 1.3	5.6
Primulanin	NA	NA	–
Ardisimamilloside H	NA	NA	–
Ketoconazole	0.04	0.05	0.8
Troleandomycin	1.5 ± 0.1	0.44 ± 0.06	3.4

*The data are represented as mean ± SD of 3 independent experiments (n = 2 in each experiment). NT = Not Tested.*

### P-gp INHIBITION

**FIGURE 5:**
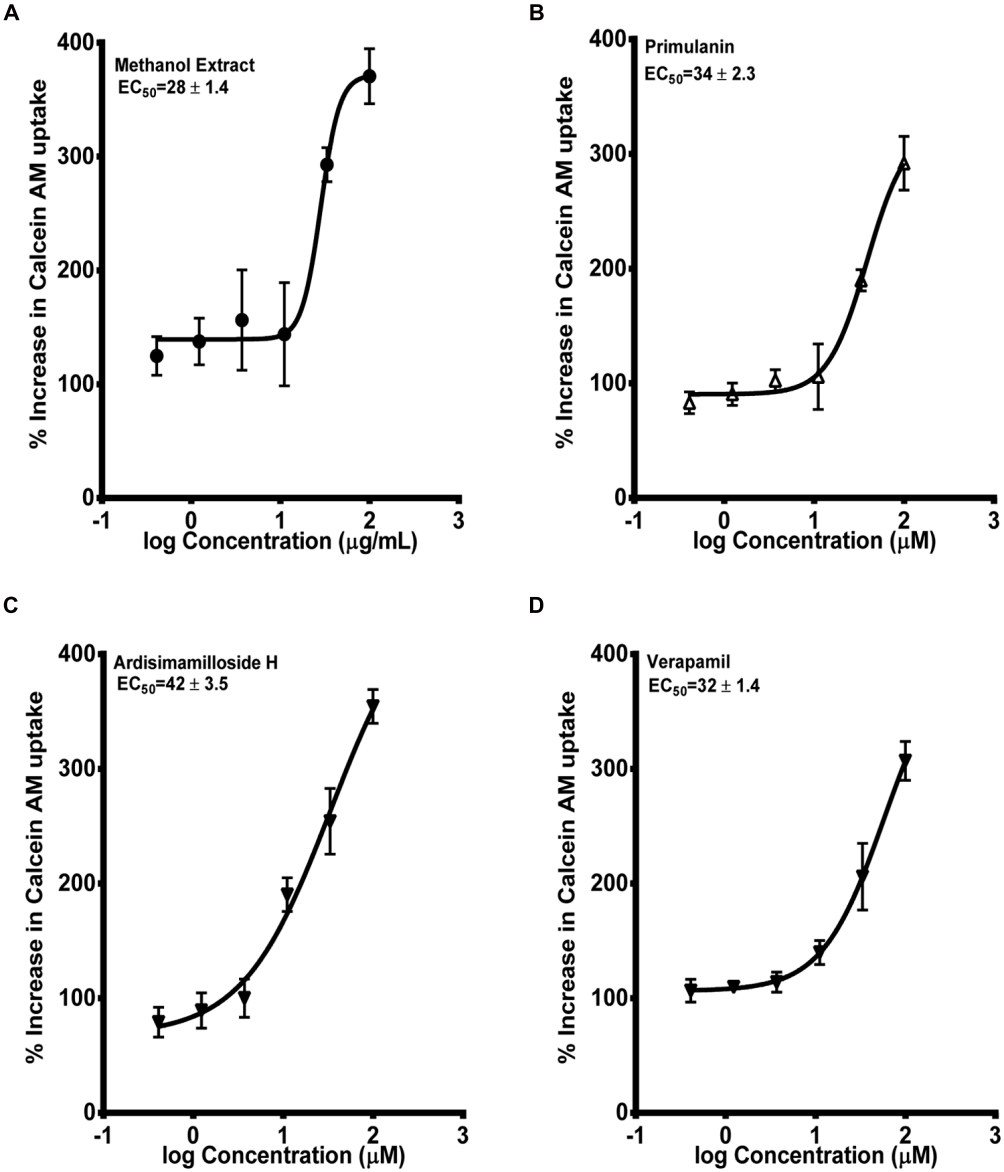
**Dose-response curves of P-gp inhibition by methanol extract of* Labisia pumila* roots **(A)**, its two saponin constituents **(B–C)** and positive control verapamil **(D)**, determined by calculating the percent uptake of calcein AM into hMDR1-MDCKII cells.** Equations used in evaluating EC_50_ and % increase in uptake of calcein-AM were described in “Materials and Methods” section. The data are represented as mean ± SD of 3 independent experiments (*n* = 2 in each experiment).

Next, we determined the inhibition of P-gp by the extract of *L. pumila* and the constituents by using the two widely used probes calcein-AM and digoxin (Rautio et al., 2006). Calcein-AM uptake was quantified in MDCK and hMDR1-MDCKII cells. The alkyl phenol compounds showed no increase in the uptake of calcein-AM in hMDR1-MDCKII cells. The methanolic extract and the two saponins, primulanin, and ardisimamilloside H, increased the uptake of calcein-AM dose dependently with EC_50_ values of 28 ± 1.4 μg/mL and 34 ± 2.3 and 42 ± 3.5 μM, respectively, as shown in **Figure 5**. The effect is comparable to the effect of positive control, verapamil (EC_50_ 32 ± 1.4 μM) but significantly less potent than the effect of cyclosporin A (EC_50_ 8 ± 1.2 μM). The P_app_ value of LY was in the range of 1.1 ± 0.8 × 10^-6^ cm/s, which was similar to our previously published values (Manda et al., 2013). Additionally, the TEER measurements before and after experiments confirmed that the test compounds did not alter the monolayer integrity during the experiment.

**FIGURE 6:**
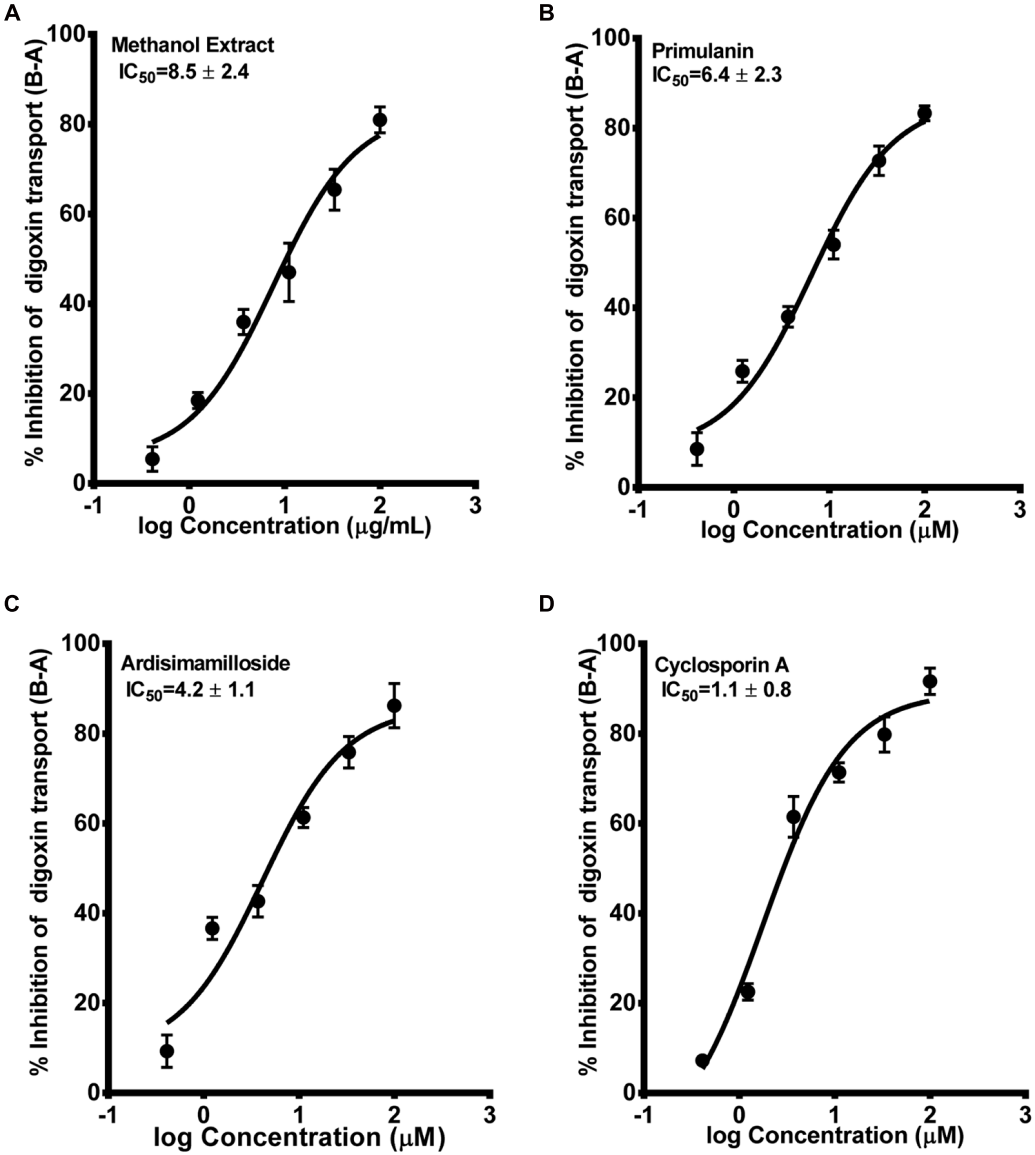
**Dose-response curves of P-gp inhibition by methanol extract **(A)** of* Labisia pumila* roots, its two saponin constituents **(B–C)** and positive control cyclosporin A **(D)**, determined by calculating the basolateral to apical transport (%) of ^**3**^H-digoxin across hMDR1-MDCKII cell monolayers.** The data are represented as mean ± SD of 3 independent experiments (*n* = 1 in each experiment).

The second probe used to determine the P-gp inhibition was radiolabelled digoxin [^3^H-digoxin]. Similar to the calcein-AM assay, the alkyl phenols had no effect on the basal to apical transport of digoxin in hMDR1-MDCKII cell monolayers, while the saponins and methanol extract showed strong inhibition. The IC_50_ values for primulanin, ardisimamilloside H, and methanol extract were 6.4 ± 2.3 μM, 4.2 ± 1.1 μM, and 8.5 ± 2.4 μg/mL, respectively, as compared to 1.1 ± 0.8 μM for cyclosporin A and 12 ± 2.1 μM for verapamil as shown in **Figure 6**. These results indicated that the extract of *L. pumila* and the two saponins inhibit P-gp strongly in terms of digoxin transport as compared to calcein-AM transport suggesting that these saponins may bind to the similar binding site as for digoxin.

### PXR MODULATION

**FIGURE 7:**
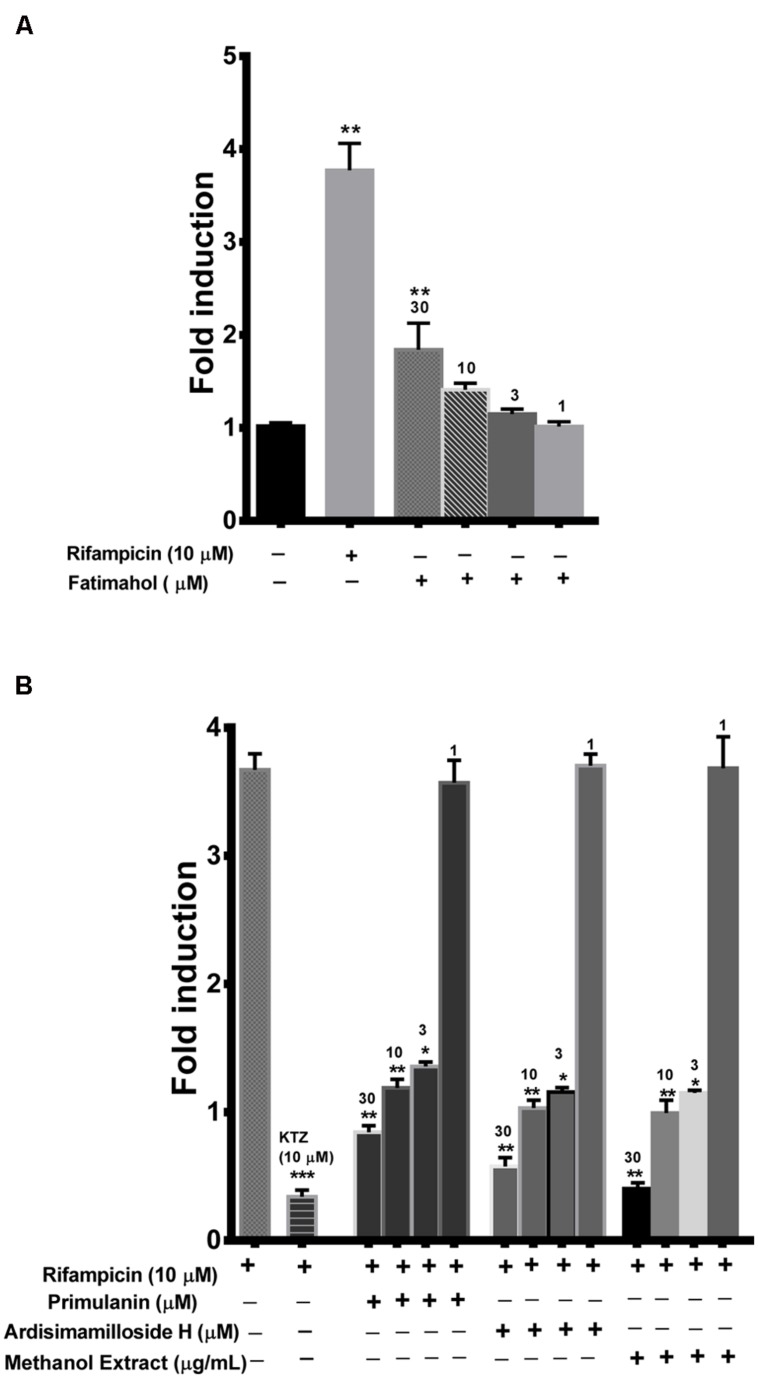
**Induction of PXR by fatimahol and rifampicin **(A)** and inhibition of rifampicin mediated induction of PXR by *Labisia pumila* methanol extract and its constituents **(B)** at indicated concentrations. Ketoconazole (KTZ) was used as a positive control in the inhibition assay.** *P < 0.05, **P < 0.01, and ***P < 0.001, determined by One way ANOVA, followed by Dunnett’s multiple comparison tests. The data are represented as mean ± SD of triplicate measurements in three independent experiments.

Finally, we looked at the modulation of PXR activity by the extract and the constituents using a reporter gene assay in HepG2 cells. One of the alky phenols, fatimahol, significantly induced PXR activity (1.8-fold) at the highest tested concentration of 30 μM, while at lower concentration the effect was not significant **Figure 7A**. These results suggest that there is no effect on the PXR activation by KF extract or its constituents. The positive control, rifampicin (10 μM) caused a fourfold induction in PXR activity which is in agreement with previous reports (Li and Chiang, 2006; **Figure 7A**). On the other hand, the methanolic extract (3–30 μg/mL) and the two saponins (primulanin and ardisimamilloside H; 3–30 μM) dose dependently decreased rifampicin-induced PXR activity (**Figure 7B**). These results indicate that *L. pumila* and its constituents significantly modulate the activity of PXR and thereby could affect the downstream genes involved in PXR signaling. Additionally, no cytotoxicity was observed toward HepG2 cells with either KF methanolic extract or its constituents up to the highest tested concentration of 30 μg/mL or 30 μM (data not shown) confirming that the inhibition of PXR as seen with methanolic extract and the two saponins is not due to the toxicity toward HepG2 cells.

### PREDICTION OF *IN VIVO* HDI FROM *IN VITRO* RESULTS

The calculated % yield and concentration per dose (560 mg, single dose) was more for alkyl phenols compared to saponins from KF roots as shown in **Table 2**. Since the intestinal absorption or plasma concentrations are not known for the test compounds, we made an assumption that all of the compounds are completely absorbed (100% bioavailable) from the GI tract. Based on this, the predicted GI and plasma concentrations of the compounds were calculated as shown in **Tables 2 and 3**. The IC_50_ values from *in vitro* CYP inhibition assays suggest that the methanolic extract of KF is likely to cause *in vivo* inhibition of all the CYPs tested and thereby potentially causing HDI (**Table 3**). All alkyl phenols except fatimahol are predicted to have a likely *in vivo* effect toward CYP2C9 and 2C19 (**Table 3**). As show in **Table 3**, the extract or the compounds did not show any strong inhibition of CYP2D6 and CYP1A2. Accordingly, we did not further determine the time-dependent inhibition (TDI) of these two enzymes.

**Table 2:** Calculation of estimated extract per dose, GIT and plasma concentrations of KF methanol extract and its constituents.

	Yield (W/W %)	Estimated concentration per dose (mg/mL)	Estimated GIT concentration (μg/mL)	Estimated Plasma concentration (μg/mL)
Methanol Extract	9.52	53.3	213	17.7
Resorcinol	1.21	6.72	27.1	2.2
Belamcandol	0.61	3.41	13.64	1.13
Demethylbelamcandaquinone B	1.21	6.77	27.1	2.25
Fatimol	0.009	0.05	0.20	0.016
Primulanin	0.014	0.07	0.31	0.026
Ardisimamilloside H	0.002	0.011	0.047	0.003

*All calculations were made based on the dose of 560 mg per day of KF extract and its constituents.*

**Table 3:** Prediction of KF methanol extract and its constituents to cause herb drug interaction *in vivo* based on *in vitro* data.

	IC_50_ (μg/mL)	GIT concentration (μg/mL)	Plasma concentration (μg/mL)	Likelihood of causing HDI
**CYP2D6**			
Methanol extract	40	213	10.65	likely
Resorcinol	28	27.1	1.46	Remote
Belamcandol	35	13.64	0.65	unlikely
Demethylbelamcandaquinone B	22	27.1	1.64	unlikely
Fatimahol	NA	0.20	0.016	unlikely
**CYP1A2**			
Methanol extract	70	213	6.086	likely
Resorcinol	29	27.1	0.774	Remote
Belamcandol	73	13.64	0.310	unlikely
Demethylbelamcandaquinone B	50	27.1	0.722	unlikely
Fatimahol	31	0.20	0.016	unlikely
**CYP3A4**			
Methanol extract	5.2	213	81.93	likely
Resorcinol	1.4	27.1	13.22	likely
Belamcandol	15	13.64	1.52	Remote
Demethylbelamcandaquinone B	45	27.1	0.79	unlikely
Fatimahol	16	0.20	0.016	unlikely
**CYP2C9**			
Methanolextract	23	213	81.93	likely
Resorcinol	3.8	27.1	13.22	likely
Belamcandol	23	13.64	1.52	remote
Demethylbelamcandaquinone B	10	27.1	0.79	likely
Fatimahol	11	0.20	0.016	unlikely
**CYP2C19**			
Methanol extract	8	213	81.93	likely
Resorcinol	1.2	27.1	13.22	likely
Belamcandol	2.2	13.64	1.52	likely
Demethylbelamcandaquinone B	5.3	27.1	0.79	likely
Fatimahol	11	0.20	0.016	unlikely

## DISCUSSION

There is increasing evidence that the global use of herbal supplements for the treatment of a wide spectrum of ailments has been on the rise. Concurrently, there are more clinical cases of toxicity caused by concomitant administration of herbal supplements with conventional medicines (Colalto, 2010). This risk is mainly attributed due to the lack of research on the drug interaction potential of herbal medicines and their constituents. Such studies are needed to identify the potential herbs which may cause drug interactions and may help in reducing the risk of clinical toxicity (Chen et al., 2012). Furthermore, it is also of significant value to identify the constituents responsible for causing drug interactions. This will also enable us to identify the potential herbs which have similar chemical composition that may cause drug interactions. Based on the FDA guidelines related to the drug interaction, identifying compounds which interact with drug metabolizing enzymes and eﬄux transporters are of paramount importance since they play a major role in altering the pharmacokinetics and pharmacodynamics of majority of conventional drugs (Huang et al., 2008). Accordingly, the current study is focused on studying a widely used herb KF and some of its chemical constituents for the possibility of herb-drug interaction mediated by modulating the activities of CYPs, P-gp, and PXR using *in vitro* methods.

In the present study, the methanolic extract of KF and its constituents showed a moderate inhibition of CYP2D6 and 1A2 at higher concentrations. It is unlikely that most of these compounds will accumulate to such high physiological concentrations after oral intake. However, saponins isolated from *Panax notoginseng* were found to induce CYP1A2 with no inhibitory effect on the activity of other CYPs (Liu et al., 2012). In contrast, we observed a strong inhibition of CYP3A4, 2C19, and 2C9 by saponins and methanol extract of KF. In a previous study, crude extracts of KF have been reported to inhibit CYP2C isoforms; however, minimal effect was seen on CYP3A4 enzyme (Pan et al., 2012). These differences toward CYP3A4 activity may be attributed to the differences in the chemical composition of the extracts used in separate studies.

Time dependent inhibitors are categorized as mechanism based inhibitors. Such inhibitors are generally considered to have more profound clinical effects compared to reversible inhibitors as they form strong covalent bonds and thereby inactivate the CYPs (Riley et al., 2007; Venkatakrishnan et al., 2007). In order to find out if the methanolic extract or the constituents cause any TDI, the shift in IC_50_ for CYP3A4, 2C9, and 2C19 was calculated as result of preincubation of samples with recombinant enzymes, NADPH, and co-factors. Neither the methanol extract nor the alkyl phenolic compounds showed TDI of CYP2C9 and 2C19 suggesting that they interact reversibly with these two enzymes. In contrast, the pre-incubation of the methanol extract and all four alkyl phenols caused a significant shift in the IC_50_ value for CYP3A4. Specifically, the IC_50_ shift for 5-[10(Z)-pentadecenyl]resorcinol, fatimahol, and the extract was greater than positive control troleandomycin, which is considered to be a clinically relevant mechanism based inhibitor. Previous structural studies have shown that the phenolic moiety strongly binds to CYP3A4 enzyme causing a strong inhibition (Stresser and Kupfer, 1997) which may explain the prominent IC_50_ shift seen with the constituents of KF in our study. Moreover, the presence of phenolic hydroxyl group has been shown to lead to potent inhibition of CYP3A4 (Ho et al., 2001). Such a phenomenon may explain the high IC_50_ shift exhibited by 5-[10(Z)-pentadecenyl]resorcinol which contains the phenolic hydroxyl group (**Figure 1**). The other alkyl phenols which did not have hydroxyl group showed comparatively weaker inhibition than 5-[10(Z)-pentadecenyl]resorcinol. This TDI of CYP3A4 could be due to either mechanism based inhibition (irreversible), or due to the generation of metabolites which may cause stronger inhibition of the enzyme. Further studies in liver microsomes are needed to clarify the exact mechanism of inhibition.

P-gp is known to contain multiple binding sites and consequently multiple probe substrates are recommended to determine if a compound is an inhibitor (Martin et al., 2000). We used calcein-AM and digoxin as our probe substrates as they are known to bind two different binding pockets of P-gp (Rautio et al., 2006). In the calcein-AM assay, the two saponins and the extract showed P-gp inhibition to a similar extent as verapamil but significantly lower than cyclosporin A. However, using digoxin as the substrate, much more potent inhibition of P-gp was seen. A similar trend was seen with verapamil which showed more potency in the digoxin assay, in accordance to previous report (Rautio et al., 2006). Our results indicate that the components of KF exhibit similar mechanism of P-gp inhibition as verapamil. Compounds which show IC_50_ values below 10 μM in the digoxin transport assay are recommended for further evaluation in the *in vivo* system (Giacomini et al., 2010). Hence the extract and constituents of KF reported in this study would meet this criterion. Various saponin-containing herbs have been shown to be potent inhibitors of P-gp (Choi et al., 2003; Doligalska et al., 2011). However, the *in vivo* efficacy depends on additional factors such as the dose and the absorption/distribution profile of an inhibitor. KF extract and its constituents showed no significant activation of PXR activity except a moderate activation by fatimahol. However, this activation was only seen at higher concentrations, which would seem unlikely to cause *in vivo* effects. Similar to P-gp inhibition, the two saponins and methanol extract showed strong inhibition of rifampicin-mediated induction of PXR activity. This may lead to a decrease in the expression of CYPs or eﬄux transporters. These effects were not due to the cytotoxicity of the KF methanol extract or its constituents as confirmed by MTS proliferation assay. Further studies are underway in our lab to determine the changes in expression of specific CYPs and eﬄux transporters using RT-PCR analysis. Based on the clinical dose of KF capsules used by the general population, we predicted the ability of the constituents and extract to cause *in vivo* interactions. Alkyl phenolic compounds and methanolic extract are likely to cause HDI. However, it is highly unlikely that the whole amount of administered herbal preparation is absorbed and is available for interaction with the drug metabolizing CYPs. Further *in vivo* studies are warranted.

In conclusion, this study demonstrated that the methanolic extract of KF and its constituents strongly inhibited (TDI) a major drug metabolizing enzyme CYP3A4, while a moderate reversible inhibition was seen with CYP2C9 and 2C19 with minimal effects on CYP 2D6 and 1A2. Inhibition of P-gp and PXR by the methanol extract could be attributed to the presence of saponins while inhibition of CYPs could be due to alkyl phenols. Taken together, concomitant use of *L. pumila* (KF) with conventional drugs could cause a possibility of drug-herb interaction. Further studies are warranted in this direction.

## Conflict of Interest Statement

The authors declare that the research was conducted in the absence of any commercial or financial relationships that could be construed as a potential conflict of interest.

## ABBREVIATIONS

CYPs: Cytochrome P450 enzymes
FDA: Food and Drug Administration
HepG2: human hepatocellular carcinoma
hMDR1-MDCK-II: human multidrug resistance-1-transfected Madin-Darby canine kidney
MDCK-II: Madin-Darby canine kidney
Ly: Lucifer yellow
P-gp: P-glycoprotein
PXR: Pregnane X receptor
TDI: Time dependent inhibition.
